# Quantum Propensity in Economics

**DOI:** 10.3389/frai.2021.772294

**Published:** 2022-01-14

**Authors:** David Orrell, Monireh Houshmand

**Affiliations:** ^1^ Systems Forecasting, Toronto, ON, Canada; ^2^ Department of Electrical Engineering, Imam Reza International University, Mashhad, Iran

**Keywords:** quantum economics, quantum finance, quantum cognition, quantum probability, quantum decision theory, quantum computing, quantum artificial intelligence

## Abstract

This paper describes an approach to economics that is inspired by quantum computing, and is motivated by the need to develop a consistent quantum mathematical framework for economics. The traditional neoclassical approach assumes that rational utility-optimisers drive market prices to a stable equilibrium, subject to external perturbations or market failures. While this approach has been highly influential, it has come under increasing criticism following the financial crisis of 2007/8. The quantum approach, in contrast, is inherently probabilistic and dynamic. Decision-makers are described, not by a utility function, but by a propensity function which specifies the probability of transacting. We show how a number of cognitive phenomena such as preference reversal and the disjunction effect can be modelled by using a simple quantum circuit to generate an appropriate propensity function. Conversely, a general propensity function can be quantized, *via* an entropic force, to incorporate effects such as interference and entanglement that characterise human decision-making. Applications to some common problems and topics in economics and finance, including the use of quantum artificial intelligence, are discussed.

## Introduction

Theories of economics always rely on theories of value. In classical economics, it was assumed that value is the product of labour (Smith, 1776). Neoclassical economists later substituted labour with the energy-like concept of utility (Jevons, 1957). In their *Theory of Games and Economic Behaviour* (1944), Von Neumann and Morgenstern (1944) developed a consistent set of axioms to describe rational economic behaviour, and the assumption that people act rationally to optimise their own expected utility became the basis for economics as it developed in the post-war era.

However while this model of rational economic behaviour remains the default approach in economics, cognitive psychologists have shown that its assumptions are often violated. For example, one of the key axioms of expected utility theory is that people have fixed preferences. Yet the widely-demonstrated phenomenon of preference reversal shows that in fact people do not have stable preferences and have a tendency to change their mind depending on things like context (Tversky and Thaler, 1990).

The belief that rational utility-optimisers drive prices to a stable equilibrium was also sorely tested by the financial crisis of 2007/8. In response to that crisis, economists began to adopt methods from behavioural economics, in which so-called cognitive anomalies were accommodated by modifying the utility function to account for effects such as loss aversion or herd behaviour (Kahneman, 2011). As discussed below, though, a range of cognitive and financial phenomena continue to elude behavioural approaches, because they do not conform to classical logic (Wendt, 2015). This has motivated interest in adopting a mathematical framework based on quantum probability.

Quantum probability is a set of mathematical rules to calculate probabilities of events in quantum mechanics. Its properties such as nonadditivity and noncommutativity make it well-suited to model uncertainty in decision-making behavior in social sciences, where it extends classical utility theory (Qadir, 1978; Segal and Segal, 1998; Baaquie, 2004; Derman, 2004; Yukalov and Sornette, 2008; Busemeyer and Bruza, 2012; Haven and Khrennikov, 2013; Khrennikov et al., 2018; Zhang and Kjellström, 2021). It also applies particularly well to the topic of money, whose function it is to collapse the fuzzy concept of value down to a number, in a manner that can be modelled as a form of wave function collapse (Orrell and Chlupatý, 2016; Orrell, 2020b). The approach in this paper is to generalize the quantum approach, by modelling economic decision-making using a probabilistic propensity function that can be expressed in quantum terms *via* the use of an entropic force. In other words, we model the economy in a manner consistent with, and inspired, by quantum computing (Nielsen and Chuang, 2002; Nakahara and Ohmi, 2008).

The plan of the remainder of the paper is as follows. *Quantum Probability* motivates the use of quantum probability to model economic decisions. *Quantum Circuits* shows how a simple quantum circuit, of a sort commonly used in quantum algorithms, can simulate a variety of cognitive phenomena which elude a classical approach. *Propensity and Entropic Force* shows how a general propensity function can be quantized to yield the quantum dynamics of financial transactions. Finally *Conclusion* summarises the main results.

## Quantum Probability

The key difference between quantum computers and classical computers is that, instead of storing information in binary bits which can take on the value 0 and 1, quantum computers use qubits, which randomly collapse to a particular state when measured. Quantum computers are therefore inherently probabilistic rather than deterministic, so a quantum circuit may have to be run and measured many times in order to build up a statistical estimate to a solution.

**FIGURE 1:**
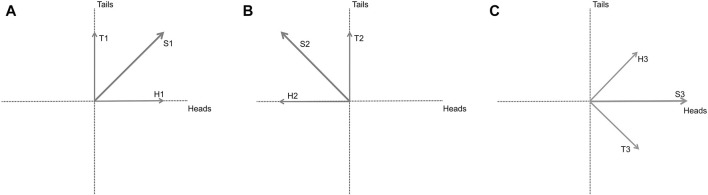
**(A)** A coin toss for a balanced coin can be expressed as a superposition of two states, heads and tails. **(B)** because the 2-norm of a probability is its square, we can also consider negative projections. **(C)** Applying the Hadamard transformation rotates S1 by 45 degrees clockwise which aligns with the H axis (S3).

In order to motivate, from first principles, the use of quantum probability in an economic context, suppose that we wish to model the state of a person who is going to make a binary choice between two options. If the person is equally likely to choose either, then the situation is the same as for a random coin toss. In general, the state could be modelled by the diagonal ray in Figure 1A, where two dimensions are required because we want to capture the possibility of either outcome. If the ray has length 1, and we associate the probabilities of obtaining heads or tails by taking the square, i.e. the 2-norm, of the projections onto the respective axis, then the probabilities add to 1 as expected.

The state can therefore be interpreted as a propensity to give different outcomes, in this case heads or tails. Because the 2-norm of a probability depends on the square, one can also imagine cases where the projections are negative (Haug, 2004; Aaronson, 2013). For example, in Figure 1B the projection on the heads axis is negative, but the 2-norm of the probability is unchanged. The fact that projections can have opposite signs allows for the possibility of interference, where probabilities cancel out instead of adding in the usual way. Since negative numbers are allowed, the need for mathematical closure suggests that complex numbers should be as well, for example to accommodate situations where we need to calculate square roots (Aaronson, 2013).

We can also consider unitary transformations which act on the state while preserving its norm. An example is the Hadamard transformation, which here rotates S1 by 45 degrees clockwise. A coin in the superposed state of Figure 1A will then be rotated so that it aligns with the H axis, as in Figure 1C, which means that when measured it will be heads for sure. The vertical component of the rays H3 and T3 have canceled out, which is an example of interference, and the indeterminate system has become deterministic.

Finally, we can also ask what happens when we have a more complicated system, for example two coins instead of one. The possible final outcomes are then HH, HT, TH, and TT, where HT is heads for the first coin and tails for the second, and so on. But if say the system is in a balanced superposition of HH and TT—i.e., there is an equal probability of getting either both heads, or both tails, and those are the only possibilities—then we can say that the coins are entangled. Entanglement can therefore be viewed as a particular kind of superposed state, which leads to correlation in measurement probabilities of subsystems.

The adoption of the 2-norm as a probabilistic measure therefore incorporates the related phenomena of superposition, interference, and entanglement, which are characteristic of quantum systems, and are exploited in quantum computers to give significant computational advantages over classical computers (Aaronson, 2013). In a quantum computer, the coin toss would be modelled using the wave function |ψ of a single qubit, which is in a superposition of two basis states $|0\rangle =(\begin{array}{c} 1\\ 0 \end{array})$ and $|1\rangle =(\begin{array}{c} 0\\ 1 \end{array})$ , with the first representing heads and the second representing tails. We can then write

$$|\psi \rangle =a_{0}|0\rangle +a_{1}|1\rangle =\left(\begin{array}{c} a_{0}\\ a_{1} \end{array}\right)$$

where $a_{0}$ and $a_{1}$ are complex numbers with ${\left|a_{0}\right|}^{2}+{\left|a_{1}\right|}^{2}=1$ . When measured in the computational basis, the possible outcomes are |0〉 with a probability ${\left|a_{0}\right|}^{2}$ and |1〉 with a probability ${\left|a_{1}\right|}^{2}$ . Quantum gates which produce transformations are represented by unitary matrices, and two or more qubits are represented by tensor products, as seen below.

To summarise, classical probability is the simplest kind of probability, which is based on the 1-norm and involves positive numbers. The next-simplest kind of probability uses the 2-norm, and includes complex numbers. The reason this kind of probability is called quantum probability, is for the historical reason that it turns out to be the right framework for quantum physics, and is the basis of quantum computing; however there is no reason we can’t apply it to other areas, such as economics.

## Quantum Circuits

Quantum probability has been adopted in areas such as quantum cognition and quantum game theory because it naturally accounts for effects such as interference and entanglement (Busemeyer and Bruza, 2012; Wendt, 2015). Well-known examples from the quantum cognition literature include the order effect, where responses to questions in a survey depend on the order in which they are asked; the disjunction effect, where extra information seems to interfere with decision-making in a manner that eludes classical logic; preference reversal, where a decision changes depending on context; or games such as the prisoner’s dilemma, where experiments show that people behave not as individual utility-optimisers, but as people who are entangled through things like a social contract.

**FIGURE 2:**
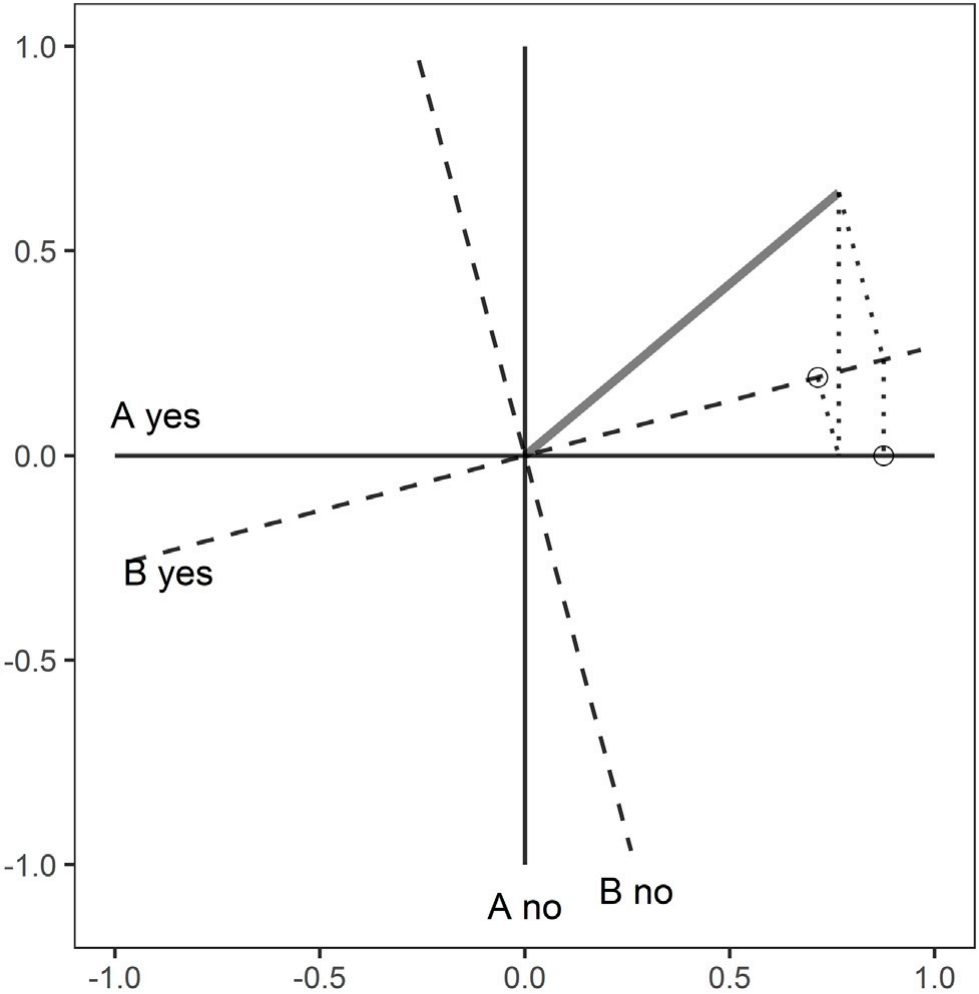
The order effect for two questions labelled A and B. The state vector (grey line) is at an angle θ to the axes for A. The axes for B are rotated by an angle φ to those for A.

For the order effect, the usual way to think about this is in terms of a sequence of projections, as illustrated in Figure 2. If question A is followed by question B, and we assume that the questions have yes/no responses, then the first response is modelled by collapsing the state, shown by the diagonal grey line, onto one of the two axes labelled “A yes” or “A no”. That state is then used as the starting point for a projection onto the B axes. Similar calculations can be made with the order reversed to reveal the order effect, which has been demonstrated in a broad range of empirical studies (Wang et al., 2014). The probability of outcomes when question A is followed by question B and vice versa are shown in Table 1.

**TABLE 1:** Probabilities of the possible outcomes in the order effect model.

Question order	A Yes	A No	B Yes	B No
A then B	$\cos^{2}\theta$	$\sin^{2}\theta$	$\cos^{2}\theta \cos^{2}\varphi +\sin^{2}\theta \sin^{2}\varphi$	$\cos^{2}\theta \sin^{2}\varphi +\sin^{2}\theta \cos^{2}\varphi$
B then A	$\cos^{2}\left(\theta -\varphi \right)\cos^{2}\varphi +\sin^{2}\left(\theta -\varphi \right)\sin^{2}\varphi$	$\cos^{2}\left(\theta -\varphi \right)\sin^{2}\varphi +\sin^{2}\left(\theta -\varphi \right)\cos^{2}\varphi$	$\cos^{2}\left(\theta -\varphi \right)$	$\sin^{2}\left(\theta -\varphi \right)$

**FIGURE 3:**
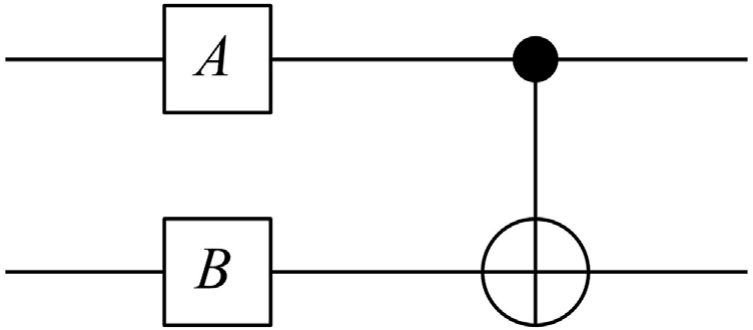
Quantum circuit for a decision *B* influenced by a context *A*.

An equivalent representation of this sequence, based on the methods of quantum computing, is the quantum circuit shown in Figure 3. The input on the left is two qubits, each of which is initialised to |0〉 . The joint initial state is written

$$\psi_{0}=|00\rangle =\left(\begin{array}{c} 1\\ 0 \end{array}\right)⊗\left(\begin{array}{c} 1\\ 0 \end{array}\right)=\left(\begin{array}{c} \begin{array}{c} 1\\ 0 \end{array}\\ 0\\ 0 \end{array}\right).$$

The top qubit is acted on by the gate *A*, which is a rotation matrix of the form

$$A=R_{\theta }=\left(\begin{array}{cc} \cos\theta & -\sin\theta \\ \sin\theta & \cos\theta \end{array}\right).$$

The lower qubit is acted on by the similar rotation matrix $B=R_{\varphi }$ where φ represents the difference in the frameworks used to answer the two questions. The two qubits are then entangled through a C-NOT gate, which is represented by the matrix

$$X_{c}=\left(\begin{array}{c} \begin{array}{c} \begin{array}{cc} 1 & \begin{array}{ccc} 0 & 0 & 0 \end{array} \end{array}\\ \begin{array}{cc} 0 & \begin{array}{ccc} 1 & 0 & 0 \end{array} \end{array} \end{array}\\ \begin{array}{cc} 0 & \begin{array}{ccc} 0 & 0 & 1 \end{array} \end{array}\\ \begin{array}{cc} 0 & \begin{array}{ccc} 0 & 1 & 0 \end{array} \end{array} \end{array}\right).$$

The operation $\psi_{f}=X_{c}\left(A⊗B\right)\psi_{0}$ depicted in Figure 3 then yields the output probabilities for the possible states shown in Table 2. Note that the total probabilities of obtaining B yes or no are the same as in Table 1 for the case where question A is followed by B . Similar calculations can be made with the order of questions reversed, by inverting the C-NOT gate so that the second qubit acts as the control and updating A as $R_{\varphi }$ and B as $R_{\left(\theta -\varphi \right)}$ .

**TABLE 2:** Probabilistic outcomes from the quantum circuit for a sequence of two queries A and then B.

Measured state	Response A	Probability	Response B	Conditional Probability	Joint Probability
|00〉	Yes	$\cos^{2}\theta$	Yes	$\cos^{2}\varphi$	$\cos^{2}\theta \cos^{2}\varphi$
|01〉	Yes	$\cos^{2}\theta$	No	$\sin^{2}\varphi$	$\cos^{2}\theta \sin^{2}\varphi$
|10〉	No	$\sin^{2}\theta$	Yes	$\sin^{2}\varphi$	$\sin^{2}\theta \sin^{2}\varphi$
|11〉	No	$\sin^{2}\theta$	No	$\cos^{2}\varphi$	$\sin^{2}\theta \cos^{2}\varphi$

As seen in the Appendix, this relationship holds when gates *A* and *B* are arbitrary unitary matrices, so the circuit is quite flexible and can be used to simulate any problem that can be described either by a sequence of projections, as in quantum cognition, or by an entangled system as in the approach known as quantum decision theory (Yukalov and Sornette, 2014; 2017). For the disjunction effect (Blutner and Graben, 2016), gate *A* represents a context or a piece of information, while gate *B* represents a decision. For preference reversal (Yukalov and Sornette, 2015), gate *A* represents a subjective context, while gate *B* represents an objective utility such as a monetary award. The circuit can also be used to simulate a version of the prisoner’s dilemma (Khan et al., 2018), where gate *B* represents one player’s strategy, and gate *A* represents their subjective ideas about the other player’s strategy (Orrell, 2021c).

A particularly strong, and economically relevant, example of a quantum social phenomenon is the existence of threshold effects (Orrell, 2021b). For the case of preference reversal, if the context-dependent subjective factors represented by gate *A* are assumed to be random, then they can be expected to have a roughly equal effect as the objective factors (such as the prize in a lottery) represented by gate *B*. Because changes in context often have a switch-like nature (for example, a person may or may not have a particular piece of information or experience a particular event) the result is a threshold effect, where objective costs must change by a set amout to overcome subjective factors. According to the preference reversal criterion (Yukalov and Sornette, 2018), if the more attractive option has an associated cost $x_{1}$ and the less attractive option has a cost $x_{2}$ , then a switch from the more attractive to the less attractive option will only occur if $x_{1}/x_{2}>3$ .

This criterion has been empirically tested in a range of experiments, and appears to be quite robust (Yukalov and Sornette, 2015; 2018). A related phenomenon is the endowment effect, where people assign a higher value to an object that they own than to one that they do not, and the switch in context from selling to buying results in a similar price gap (Kahneman et al., 1990). We return to the question of threshold effects below.

## Propensity and Entropic Force

The above examples show that the quantum approach, native to quantum computers, is well-suited to studying a variety of problems that involve interference and entanglement, and are therefore not easily addressed using classical logic or behavioural approaches, which is why quantum methods are seeing increasing use in the social sciences (Wendt, 2015; Der Derian and Wendt, 2020). For the case of economics the quantum approach, with its change from utility to propensity, leads to a shift in our understanding of how decisions are made, and how financial transactions are modelled. One thing that distinguishes economics from the other social sciences is that it involves financial transactions, so price can be used as a measure of position. We can therefore build models that use price as an independent variable. Instead of assuming that supply and demand determine price, as in neoclassical economics, we assume that price determines propensity. The fact that price is just a number, as opposed to something real and immutable, is exactly what introduces the probabilistic uncertainty that makes the quantum approach suitable.

Smith (1776) argued that the “propensity to truck, barter, and exchange” was inherent in human nature—and while we can’t directly observe utility, we can certainly observe people buying and selling. We can therefore define a propensity function as a kind of schedule which describes the probability that a person will take a certain decision. Similar propensity curves are used in marketing, where the technique known as “propensity modelling” is used to simulate how a customer’s willingness to buy is affected by attributes including price (Wilcox, 2021). Since as we have seen quantum probability can be used to derive a propensity function for the discrete case, a natural question to ask is whether it is possible to go the other way, and use a given propensity function to derive a quantum model.

**FIGURE 4:**
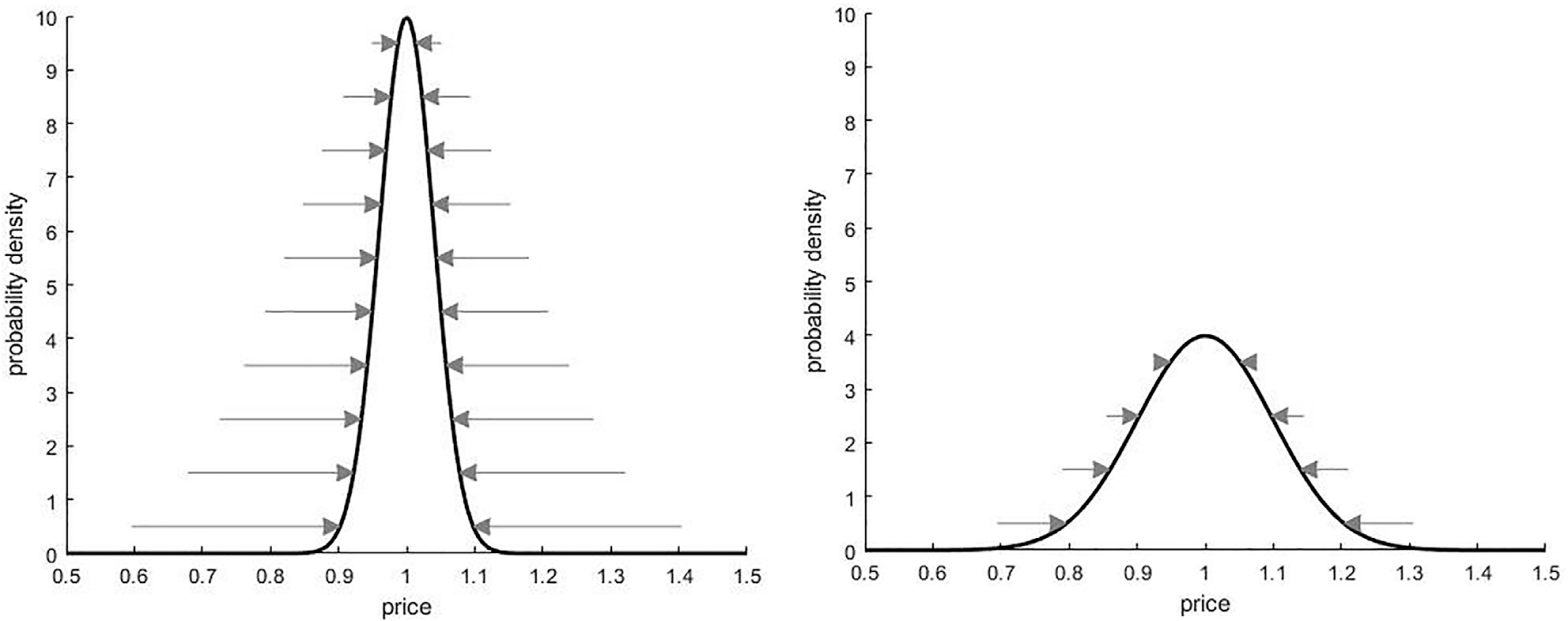
The curves show propensity as a function of price, measured in millions of dollars. Both are centered at *p* = 1, but the panel on the right has a higher level of price flexibility. The arrows indicate the strength and direction of the associated entropic forces (discussed later).

As an example, suppose that we made a probabilistic estimate of the price of a house. The result could resemble a normal distribution, as in Figure 4, where the center of the distribution would be our best estimate, while the standard deviation would be a measure of our uncertainty. In quantum terms, this can be interpreted as a superposition state, where the chance of selecting a particular price depends on the squared amplitude of an underlying wave function. The situation is therefore similar to the coin toss, except that instead of only heads or tails there is now a continuous range of possible outcomes.

While it is traditional in economics to talk about the forces of supply and demand, these forces are assumed to cancel at equilibrium, and there is no consistent concept of economic mass. The dynamics of economic transactions are therefore not usually considered in detail, other than to assume the system is at balance. In contrast, an advantage of the propensity framework is that it leads to the concept of entropic force, which reflects the tendency of a system to achieve maximum entropy (Sokolov, 2010; Caticha, 2019). In statistical physics, an entropic force for a probability distribution P(x) is given by

$$f\left(x\right)=\gamma \frac{P\prime \left(x\right)}{P\left(x\right)}=k_{B}T\frac{P\prime \left(x\right)}{P\left(x\right)}$$

where T is temperature and $k_{B}$ is the Boltzmann constant. In economics, the propensity can similarly be viewed as the product of an entropic force acting on the mental state of the buyer/seller; and γ can be interpreted as a kind of energy which is related to information (Orrell, 2020a).

In the case that the propensity function P(x) is normal with mean μ and standard deviation  σ , where price x is a logarithmic variable, then the corresponding entropic force is

$$F\left(x\right)=\gamma \frac{P\prime \left(x\right)}{P\left(x\right)}=-k\left(x-\mu \right)$$

where $k=\gamma /\sigma^{2}$ is a force constant. The linear force therefore represents the mental desire for a buyer or seller to adjust the price to their own preferred level.

While the entropic force allows us to interpret the system in terms of dynamics, it doesn’t tell us anything about the relevant mass that the force acts on; and as already seen we want to be able to account for effects such as interference and entanglement. We can address these issues by again moving to a quantum framework, and viewing the propensity function as being the product of an underlying wave function. The quantum version of a linear spring system is of course the quantum harmonic oscillator, whose ground state is a normal distribution with mean μ and standard deviation σ . The associated mass is

$$m=\;\frac{\hbar }{2\omega \sigma^{2}}$$

so mass varies inversely with variance. Referring to Figure 4, the narrow propensity curve on the left has a higher associated mass than does the wider curve on the right. The scaling factor γ is given by γ=ℏω/2 which has units of energy.

In statistical physics, a frequency ω is related to the inverse of the Boltzmann time $\tau_{B}=\hbar /\left(k_{B}T\right)$ which is the theoretical order of time needed for an arbitrary nonstationary state to reach thermal equilibrium (Goldstein, Hara, and Tasaki, 2015). In the social version, the frequency can therefore be thought of as representing a linearised resistance to change. For something like a stock market, the frequency can be used to represent the speed of mean reversion of returns (Ahn et al., 2017), while the constant ℏ can be intepreted as a scaling factor.

In an economic transaction, the buyer propensity function will normally have a higher mean price than that of the seller, so the only parts of these curves which will be active are near the mid-price. It is easily checked that the joint propensity function, which is the product of the buyer and seller propensities, is a scaled normal curve and has an entropic force which is just the sum of the buyer and seller forces (Orrell, 2020a). The case where the supplier fixes the price and refuses to negotiate is handled by assuming an infinitely thin propensity curve located at the sale price.

Because the oscillator model is inherently probabilistic, it can be used to model market phenomena such as the pricing and volume of financial options (Orrell, 2021a; Anonymous, 2021) for which empirical data is readily available. Another difference between quantum and classical oscillators is that the former features discrete energy levels. The ground state, which again can be viewed as representing the potential for a transaction to occur, corresponds to the normal distribution, while the other states show more complicated distributions, and contribute the non-normal behavior also seen with markets (Ahn et al., 2017).

A main advantage of the quantum approach, when coupled with the entropic approach, is therefore that it gives us a consistent set of equations and units with which we can describe the dynamics of the system; and is particularly well adapted to the study of financial transactions, which involve the flow of information rather than just physical objects. For example, returning to the general case where the entropic force is given by

$$f\left(x\right)=\gamma \frac{P\prime \left(x\right)}{P\left(x\right)}$$

we can look at a particular mental state where the log price is $x_{1}$ and ask how much work—which again is linked to information—must be done against the entropic force to move to another state $x_{2}$ . This is simply

$$\Delta E=\int_{x_{1}}^{x_{2}}F\left(x\right)dx=\gamma \log\left(\frac{P\left(x_{2}\right)}{P\left(x_{1}\right)}\right)$$

which depends only on the ratio of the initial and final propensities. The change in propensity required for preference reversal, as mentioned above, is typically a factor 3, which is close to Euler’s number *e*. It follows that what might be called the energy required to change a person’s mind is

$$\Delta E=\frac{\hbar \omega }{2}\log\left(3\right)≅\frac{\hbar \omega }{2}\log\left(e\right)=\frac{\hbar \omega }{2}$$

which is the base energy of a quantum oscillator, thus highlighting the connection between quantum cognition and economic transactions (which occur of course as the result of individual decisions). In a quantum computer, it is also the order of energy needed to flip a qubit from one state to another.

## Conclusion

To summarise, expressing economic decisions in terms of a quantum circuit allows us to incorporate effects such as interference and entanglement; and applying the concept of entropic force, with price as an independent variable, allows us to derive a quantum economic model, complete with versions of force and energy. In early neoclassical economics, utility was viewed as a kind of energy. In his 1892 book *Mathematical Investigations in the Theory of Value and Prices*, Irving Fisher for example expressed economic transactions in physical terms, where utility had units of energy. The quantum framework returns to this idea of energy, but associates it with a change in propensity rather than a utility. Table 3 summarises some of the key differences between the classical and quantum approaches.

**TABLE 3:** Comparison of the quantum and classical approaches.

Classical	Quantum
Utility	Propensity
Probability measured using 1-norm	Probability measured using 2-norm
Fixed preferences	Superposition states
Additivity of causes	Interference, threshold effects
Independent agents	Entangled agents
Objectivity	Objectivity plus subjectivity
Forces cancel at equilibrium	Entropic forces lead to dynamics
No concept of inertial mass	Mass scales with inverse variance
Determinism	Uncertainty
Price measures value	Price gives an eigenvalue

Since the goal in artificial intelligence can be viewed as minimizing entropy, it is obviously attractive to base the modelling framework on entropy as well. It is interesting to note for example that the basic entanglement circuit depicted in Figure 3 is used as a building block in algorithms for things like genetic machine learning algorithms (Kondratyev, 2020).

As already mentioned the field of quantum cognition is based on empirical results such as the order effect and preference reversal which falsify the classical expected utility theory, so the focus in this paper has been on using these results to build a theory that applies more generally to economic transactions. The propensity approach allows us to view the economy as a quantized probabilistic system, of the sort simulated in quantum computing. Of course, many social scientists will argue that it is inappropriate to quantify things like social power or mental energy, since they cannot be reduced to exact equations, but one consequence of the rational utility-optimizing picture is that complex social topics such as power relationships were downplayed or ignored (Häring and Douglas, 2013; Orrell, 2017). Since economics is a quantitative discipline, we need a suitable framework with which to describe the interplay between objective and subjective forces that make up power.

While human motivations cannot be reduced to equations, it seems reasonable to quantify the propensity for a person to make a particular decision (indeed, this is the entire basis for fields such as behavioural economics). The entropic force (along with its associated energy) is merely another way of expressing a propensity function. The approach can be used to simulate a variety of economic phenomena, from cognitive interference in the decision-making process, to the price and trading volume of financial options. The affinity between finance and quantum computing is particularly evident in the area of quantum finance, where quantum algorithms are coming into their own (Nogueiras et al., 2021; Anonymous, 2021).

To summarise, neoclassical economics was marked by a switch from a labour theory of value, to one based on utility. Quantum economics makes a similar switch, by expressing value in terms of a propensity function. Adopting a quantum probabilistic framework allows us to incorporate cognitive effects such as interference and entanglement; express basic economic quantities such as forces of supply and demand in consistent units; and consider both subjective and objective factors on an equal footing.

## Data Availability

The original contributions presented in the study are included in the article/Supplementary Material, further inquiries can be directed to the corresponding author.

## References

[B1] Aaronson S. (2013). Quantum Computing since Democritus. Cambridge: Cambridge University Press.

[B2] Ahn K. Choi M. Y. Dai B. Sohn S. Yang B. (2017). Modeling Stock Return Distributions with a Quantum Harmonic Oscillator. EPL 120 (3), 38003. 10.1209/0295-5075/120/38003

[B3] Anonymous (2021). Schrödinger’s Markets. London: The Economist.

[B4] Baaquie B. E. (2004). Quantum Finance. Cambridge: Cambridge University Press.

[B49] Blutner R. Graben B. P. (2016). Quantum cognition and bounded rationality. Synthese 193, 3239–3291. 10.1007/s11229-015-0928-5

[B6] Busemeyer J. Bruza P. (2012). Quantum Models of Cognition and Decision. Cambridge: Cambridge University Press.

[B7] Caticha A. (2019). The Entropic Dynamics Approach to Quantum Mechanics. Entropy 21 (10), 943. 10.3390/e21100943

[B8] Der Derian J. Wendt A. (2020). 'Quantizing International Relations': The Case for Quantum Approaches to International Theory and Security Practice. Security Dialogue 51 (5), 399–413. 10.1177/0967010620901905

[B9] Derman D. (2004). My Life as a Quant: Reflections on Physics and Finance. Hoboken: Wiley.

[B11] Goldstein S. Hara T. Tasaki H. (2015). Extremely Quick Thermalization in a Macroscopic Quantum System for a Typical Nonequilibrium Subspace. New J. Phys. 17, 045002. 10.1088/1367-2630/17/4/045002

[B12] Haug E. G. (2004). Why So Negative to Negative Probabilities. London: Wilmott Magazine, 34–38.

[B13] Haven E. Khrennikov A. (2013). Quantum Social Science. Cambridge: Cambridge University Press.

[B14] Häring N. Douglas N. (2013). Economists and the Powerful: Convenient Theories, Distorted Facts, Ample Rewards. London: Anthem Press.

[B15] Jevons W. S. (1957). The Theory of Political Economy. 5th Edition. New York: Kelley and Millman.

[B16] Kahneman D. (2011). Thinking, Fast and Slow. New York: Farrar, Straus and Giroux.

[B17] Kahneman D. Knetsch J. L. Thaler R. H. (1990). Experimental Tests of the Endowment Effect and the Coase Theorem. J. Polit. Economy 98, 1325–1348. 10.1086/261737

[B18] Khan F. S. Solmeyer N. Balu R. Humble T. S. (2018). Quantum Games: a Review of the History, Current State, and Interpretation. Quan. Inf Process 17 (11), 309. 10.1007/s11128-018-2082-8

[B50] Khrennikov A. Basieva I. Pothos E. M. Yamato I. (2018). Quantum Games: a Review of the History, Current State, and Interpretation. Sci. Rep. 8, 16225. 10.1038/s41598-018-34531-3 30385809 PMC6212453

[B20] Kondratyev A. (2020). Non-Differentiable Learning of Quantum Circuit Born Machine with Genetic Algorithm. Available at: https://ssrn.com/abstract=3569226 .

[B21] Nakahara M. Ohmi T. (2008). Quantum Computing: From Linear Algebra to Physical Realizations. Boca Raton: Taylor & Francis.

[B22] Nielsen M. A. Chuang I. L. (2002). Quantum Computation and Quantum Information. Cambridge: Cambridge University Press.

[B23] Nogueiras M. Sanz G. O. Cendon C. V. Rodriguez A. L. Herrer A. M. Musso D. (2021). Review of State-Of-The-Art for Pricing and Computation of VaR. Paris: NEASQC. Available at: https://www.neasqc.eu/wp-content/uploads/2021/06/NEASQC_D5.1_Review-of-state-of-the-art-for-Pricing-and-Computation-of-VaR_R2.0_Final.pdf .

[B25] Orrell D. (2017). Economyths: 11 Ways Economics Gets it Wrong. London: Icon Books.

[B27] Orrell D. (2020a). A Quantum Model of Supply and Demand. Physica A: Stat. Mech. its Appl. 539, 122928. 10.1016/j.physa.2019.122928

[B30] Orrell D. (2020b). The Value of Value: A Quantum Approach to Economics, Security and International Relations. Security Dialogue 51 (5), 482–498. 10.1177/0967010620901910

[B28] Orrell D. (2021a). A Quantum Walk Model of Financial Options. Wilmott 2021 (112), 62–69. 10.1002/wilm.10918

[B29] Orrell D. (2021b). The Color of Money: Threshold Effects in Quantum Economics. Quan. Rep. 3 (2), 325–332. 10.3390/quantum3020020

[B26] Orrell D. (2021c). Quantum Economics and Finance: An Applied Mathematics Introduction. Second edition. New York: Panda Ohana.

[B24] Orrell D. Chlupatý R. (2016). The Evolution of Money. New York: Columbia University Press.

[B31] Qadir A. (1978). Quantum Economics. Pakistan Econ. Soc. Rev. 16 (3/4), 117–126.

[B33] Segal W. Segal I. E. (1998). The Black-Scholes Pricing Formula in the Quantum Context. Proc. Natl. Acad. Sci. 95, 4072–4075. 10.1073/pnas.95.7.4072 9520495 PMC19965

[B34] Smith A. (1776). An Inquiry into the Nature and Causes of the Wealth of Nations. London: W. Strahan & T. Cadell.

[B35] Sokolov I. M. (2010). Statistical Mechanics of Entropic Forces: Disassembling a Toy. Eur. J. Phys. 31, 1353–1367. 10.1088/0143-0807/31/6/005

[B36] Steeb W-H. (2006). Problems and Solutions in Introductory and Advanced Matrix Calculus. Singapore: World Scientific.

[B37] Tversky A. Thaler R. H. (1990). Anomalies: Preference Reversals. J. Econ. Perspect. 4, 201–211. 10.1257/jep.4.2.201

[B38] Von Neumann J. Morgenstern O. (1944). Theory of Games and Economic Behavior. Princeton, NJ: Princeton University Press.

[B39] Wang Z. Solloway T. Shiffrin R. M. Busemeyer J. R. (2014). Context Effects Produced by Question Orders Reveal Quantum Nature of Human Judgments. Proc. Natl. Acad. Sci. USA 111 (26), 9431–9436. 10.1073/pnas.1407756111 24979797 PMC4084470

[B40] Wendt A. (2015). Quantum Mind and Social Science: Unifying Physical and Social Ontology. Cambridge: Cambridge University Press.

[B41] Wilcox R. (2021). Conjoint Analysis: Propensity Modeling. Coursera Video. Available at: https://www.coursera.org/lecture/uva-darden-bcg-pricing-strategy-customer-value/conjoint-analysis-propensity-modeling-JZjcJ (Accessed March 1, 2021).

[B42] Yukalov V. I. Sornette D. (2015). Preference Reversal in Quantum Decision Theory. Front. Psychol. 6, 1538–1547. 10.3389/fpsyg.2015.01538 26500592 PMC4597272

[B43] Yukalov V. I. Sornette D. (2014). Conditions for Quantum Interference in Cognitive Sciences. Top. Cogn. Sci. 6, 79–90. 10.1111/tops.12065 24259280

[B44] Yukalov V. I. Sornette D. (2018). Quantitative Predictions in Quantum Decision Theory. IEEE Trans. Syst. Man. Cybern, Syst. 48 (3), 366–381. 10.1109/tsmc.2016.2596578

[B45] Yukalov V. I. Sornette D. (2008). Quantum Decision Theory as Quantum Theory of Measurement. Phys. Lett. A 372 (46), 6867–6871. 10.1016/j.physleta.2008.09.053

[B46] Yukalov V. I. Sornette D. (2017). Quantum Probabilities as Behavioral Probabilities. Entropy 19 (3), 112. 10.3390/e19030112

[B47] Zhang C. Kjellström H. (2021) A Subjective Model of Human Decision Making Based on Quantum Decision Theory. arXiv preprint arXiv:2101.05851.

