# Supplementary material for: Quantum Propensity in Economics
Source: Front Artif Intell. 2022 Jan 14;4:772294. doi: 10.3389/frai.2021.772294 (PMC8795949; doi:10.3389/frai.2021.772294)
Supplement: Supplementary file 1 [file Presentation1.pdf]

Appendix

Each measurement in a non-computational basis is equivalent to applying a unitary matrix and then measuring in the computational basis (Nielsen and Chuang, 2002). It follows that the circuit in Figure 2 is equivalent to Figure A.1, where $\mathrm{A} = \begin{bmatrix} \mathrm{a}_{11} & \mathrm{a}_{12} \\ \mathrm{a}_{21} & \mathrm{a}_{22} \end{bmatrix}$ and $\mathrm{B} = \begin{bmatrix} \mathrm{b}_{11} & \mathrm{b}_{12} \\ \mathrm{b}_{21} & \mathrm{b}_{22} \end{bmatrix}$ are unitary matrices determined based on the given non-computational basis. This Appendix shows how this one-qubit circuit is equivalent to the two-qubit quantum circuit depicted in Figure 3 for the general case where A and B are unitary matrices.

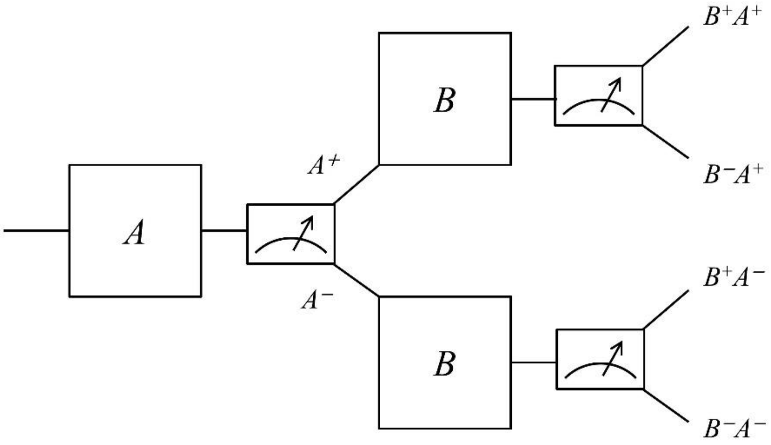

Figure A.1. A one-qubit circuit with two stages of measurement.

For the one-qubit circuit in Figure A.1, the qubit after gate A will be in the state $A|0\rangle = \begin{bmatrix} a_{11} \\ a_{21} \end{bmatrix}$. The first measurement collapses the state to $|0\rangle$ with probability equal to $|a_{11}|^2$ (EVENT A^+) and to $|1\rangle$ with probability $|a_{21}|^2$ (EVENT A^-). For the order effect, A^+ would correspond to answering “Yes” to the first question, and A^- would correspond to answering “No”.

If EVENT A^+ happens, then the output state after applying B is $B|0\rangle = \begin{bmatrix} b_{11} \\ b_{21} \end{bmatrix}$. The second measurement collapses the state to $|0\rangle$ with probability equal to $|b_{11}|^2$ (EVENT B^+) and collapses to $|1\rangle$ with probability equal to $|b_{21}|^2$ (EVENT B^-).

With the same argument for the other path we have:

EVENTS	A^+B^+	A^+B^-	A^-B^+	A^-B^-
Result State	$\|00\rangle$	$\|01\rangle$	$\|10\rangle$	$\|11\rangle$
Probability	$\|a_{11}b_{11}\|^2$	$\|a_{11}b_{21}\|^2$	$\|a_{21}b_{12}\|^2$	$\|a_{21}b_{22}\|^2$

On the other hand the output of the two-qubit circuit, when the control qubit is at the top of the circuit, is $a_{11}b_{11}|00\rangle + a_{11}b_{21}|01\rangle + a_{21}b_{21}|10\rangle + a_{21}b_{11}|11\rangle$ with associated probabilities:

Result State	$\|00\rangle$	$\|01\rangle$	$\|10\rangle$	$\|11\rangle$
Probability	$\|a_{11}b_{11}\|^2$	$\|a_{11}b_{21}\|^2$	$\|a_{21}b_{21}\|^2$	$\|a_{21}b_{11}\|^2$

Since B is unitary, its columns (or its rows) form an orthonormal basis (Steeb, 2006). In the case of a 2-by-2 unitary matrix, $|b_{11}|^2 + |b_{12}|^2 = |b_{11}|^2 + |b_{21}|^2 = 1$ which implies $|b_{12}|^2 = |b_{21}|^2$, and $|b_{11}|^2 + |b_{21}|^2 = |b_{21}|^2 + |b_{22}|^2 = 1$ which implies $|b_{11}|^2 = |b_{22}|^2$. It therefore follows that the final probabilities for the two circuits are the same. (Note that the intermediate probabilities are not the same.)
